# Study of Wear, Stress and Vibration Characteristics of Silicon Carbide Tool Inserts and Nano Multi-Layered Titanium Nitride-Coated Cutting Tool Inserts in Turning of SS304 Steels

**DOI:** 10.3390/ma15227994

**Published:** 2022-11-12

**Authors:** S. Ganeshkumar, Bipin Kumar Singh, S. Dharani Kumar, S. Gokulkumar, Shubham Sharma, Kuwar Mausam, Changhe Li, Yanbin Zhang, Elsayed Mohamed Tag Eldin

**Affiliations:** 1 Mechanical Engineering, Sri Eshwar College of Engineering, Coimbatore 641202, India; 2 Mechanical Engineering, KPR College of Engineering and Technology, Coimbatore 641407, India; 3 Mechanical Engineering Department, University Center for Research & Development, Chandigarh University, Mohali 140413, India; 4 School of Mechanical and Automotive Engineering, Qingdao University of Technology, Qingdao 266520, China; 5 Department of Mechanical Engineering, GLA University, Mathura 281406, India; 6 Faculty of Engineering and Technology, Future University in Egypt, New Cairo 11835, Egypt

**Keywords:** tool life, tool inserts, cutting forces, vibration, stress analysis

## Abstract

Cutting tool characterization plays a crucial role in understanding the behavior of machining operations. The selection of a suitable cutting material, the operating conditions for the work piece, is necessary to yield good cutting-tool life. Several pieces of research have been carried out in cutting-tool characteristics for turning operation. Only a few pieces of research have focused on correlating the vibrations and stress with wear characteristics. This research article deals with stress induced in silicon carbide tool inserts and coated tool inserts while machining SS304 steel. Since this material is much less resistant to corrosion and oxidation it is widely used in engineering applications such as cryogenics, the food industry and liquid contact surfaces. Moreover, these materials have much lower magnetic permeability so they are used as nonmagnetic engineering components which are very hard. This article focuses on the machining of SS304 by carbide tool inserts and then, the cutting forces were observed with a tool dynamometer. Using observed cutting forces, the induced stress in the lathe tool insert was determined by FEA investigation. This research also formulates an idea to predict the tool wear due to vibration. Apparently, the worn-out tool vibrates more than new tools. Using the results, the relation between stress, strain and feed rate, depth of cut and speed was found and mathematically modeled using MINI TAB. It was observed that carbide tool inserts with coating withstand better than uncoated tools while machining SS304. The results were anticipated and correlation between the machining parameters furnished the prediction of tool life and obtaining the best machining outcomes by using coated tool inserts.

## 1. Introduction

In modern machining processes, the finished product demands very high accuracy and a good surface finish with lower machining costs. In machining processes, various parameters were involved to predict the tool life, such as the feed rate, depth of cut and speed of spindle. The results can be correlated with output parameters such as surface finish, wear rate and vibrations. The life of the cutting tool depends upon the cutting speed and characteristics of material; from Taylor’s equation, the tool life can be exhibited as VT^n^ = C, where V is the cutting speed, T is tool life, C is constant, n is material constant which varies with respect to the material of tool insert, and for HSS is 0.10–0.15, carbides 0.20–0.25, and ceramics 0.6–1.0. This equation gives the relation between tool life and cutting speed. Moreover, the parameters such as depth of cut and material of work piece also predict the tool life. If the tool life was expired then the surface finish cannot be obtained and the quality of the product will decline. Hence, to improve the machining, monitoring of the accuracy and predicting the tool life are very essential. These can be completed by experimental and theoretical methods.

The cutting quality of the machining tool was investigated in the machining of steels. The flank wear and crater wear were measured in the turning tests and the correlation between the tool failure and the quality of the machining strongly influences the flank wear of the tool inserts [1]. The cutting state of an end mill was monitored and the relationship between the cutting state and the time series was investigated. The theoretical and experimental analysis exhibits the characteristics of the wear pattern of the end mill tools with the time series [2]. The tool-wear monitoring system using acoustic emission was carried out in machining of steels. The data acquisition systems and wear measuring techniques are exhibited in the research using acoustic emissions [3]. The characteristics of single layer and multilayer tool-insert coatings were reviewed for TiN Al-based materials. The review criticized the manufacturing methods of coated tool inserts such as the coating of cutting tools by vapor deposition method (CVD) and the physical vapor deposition method (PVD). The ionization rate of the coating method affects the film thickness and influences the wear behavior in testing conditions [4]. The wear mechanisms of TiN-coated and uncoated cermet tool inserts were analyzed while machining AISI H13 tool steel under high speed conditions. The wear growth was depicted and the pattern of progressive wear while machining was observed using a tool maker’s microscope and recorded; the research stated the influence of the cutting speed, feed rate and depth of cut in the progressive wear [5]. The influence of cutting edge geometry was investigated in the finish turning of AISIH13 steels. Cubic-born Nitride (CBN) tool inserts are used in dry machining conditions. Hardness, edge geometry, feed rate and cutting speed were taken into account and the wear pattern was plotted. The results from the research were analyzed statistically to find the percentage of errors. The results concluded that the tool geometry strongly influenced the wear behavior along with the hardness, feed rate and the cutting speed [6]. The experiment was carried out to state the machining behavior of Silicon Carbide tool inserts and Titanium Nitride-coated tool inserts in the machining of EN8 and EN36 materials under dry conditions. The Taguchi design of experiments was selected to formulate the combination of machining parameters, L25 orthogonal array was selected and experimental results exhibited the wear patterns of SiC and TiN-coated tool inserts. The TiN-coated tool inserts performed better than the uncoated SiC tool inserts [7,8]. The effect of tool geometry on the wear of cemented carbide tools coated with TiAlN inserts were studied while drilling compacted graphite iron (CGI) materials. The research exhibits the relevance parameters to the metallurgical properties [9]. The wear pattern of diamond tool inserts were measured during the machining Al 6061 and 1215 steel in dry conditions. The abrasive and chemical properties were studied in the research and the chemical wear of the work piece was investigated which showed the significance of the chemical behavior of the tool insert in wear patterns [10]. The investigation and finite element method (FEM)-based simulation were carried out using uncoated Silicon Carbide tool inserts. The FEM simulations were carried out using deform 3D software and the results depict the wear behavior of the Silicon Carbide tool inserts in dry machining conditions [11]. The mathematical model was derived for wear behavior in the turning process of Inconel 718 alloys. The tests were carried out in dry machining conditions and the governing equations were formulated based on the cutting forces acting on the tool insert, considering the tool holder as a cantilever beam and the deformation and stress acting on the tool inserts were simulated numerically. The data recorded in the testing are stated as a dataset and the dataset was used in data exploratory analysis [12]. A numerical study of the stress behavior of the tool inserts while machining EN8 steels was investigated with the machining of Silicon Carbide tool inserts. The results concluded that the cutting forces in x, y, and z Cartesian coordinates strongly influence the stress behavior. The linear regression equations showed the significance of stress acting on the tool inserts due to the cutting forces [13,14]. The tool condition monitoring and tool wear prediction strongly influences the quality of production and integrating these smart manufacturing methods in industry 4.0 paves the way for a reduction in wastages due to the lack of accuracy of the work piece [15]. The wear behavior of an uncoated carbide tool in machining of Ti-5553 material in cryogenic conditions under liquid nitrogen cooling was analyzed. The tool wear modes under various cutting parameters were tested. The tool wear map that was developed for the rake face was exhibited. The experimental results conclude that the relation of a flank-face tool wear map and the machining conditions leads to the selection of cutting parameters to achieve a better precision, accuracy and good surface finish of the work piece. Various pieces of research have been carried out in online tool-condition monitoring and tool prediction using machine learning techniques. The data used to teach the machine are based on these experiments. Hence, research paves the way in tool condition monitoring using machine learning algorithms. From the previous literature, there are a few studies focused on correlating the vibrational characteristics and the wear behavior. The investigation of the vibrational characteristics of Silicon carbide (SiC) tool inserts and Titanium Nitride (TiN)-coated tool inserts and the stress induced due to cutting forces are the foremost objectives of the research. The ultrasonic vibration-assisted turning technique was developed. The high frequency and low amplitude sound waves are superimposed to determine the vibrations in the conventional turning process. In addition, the research explored the relationship between the surface roughness and cutting forces in operation and the machining parameters such as speed, feed and depth of cut. The superimposed tool characteristics are determined in tangential and radial directions [16,17].

## 2. Experimentation

### 2.1. Materials and Methods

The tool wear patterns were investigated in Silicon Carbide and Titanium Nitride-coated tool insert with 25 combinations of the trial turning tests. The SS304 rod of 25.4 mm diameter and 300 mm length was used in the turning tests. The length and width of the tool holder was 125 mm and 10 mm, respectively. The design of experiments was completed using the Taguchi L25 orthogonal array derived for the combination of machining parameters. The machining parameters taken into consideration are shown in Table 1.

The cutting forces acting on the tool inserts in the x, y and z direction Cartesian coordinates were measured using a lathe tool dynamometer. Silicon carbide uncoated tool inserts and Titanium Nitride-coated tool inserts were used to machine the SS304 material in a constant volume of metal removal and a variation of the cutting forces, with feed rate, cutting speed and depth of cut recorded. The tool nomenclature for the CNMG turning tool holder is illustrated in Table 2.

**Figure 1:**
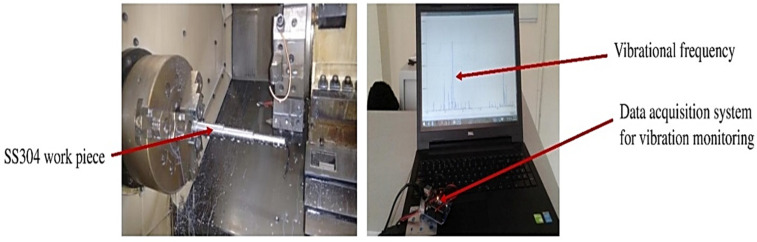
Machining of SS304 work piece with coated tool insert.

**Figure 2:**
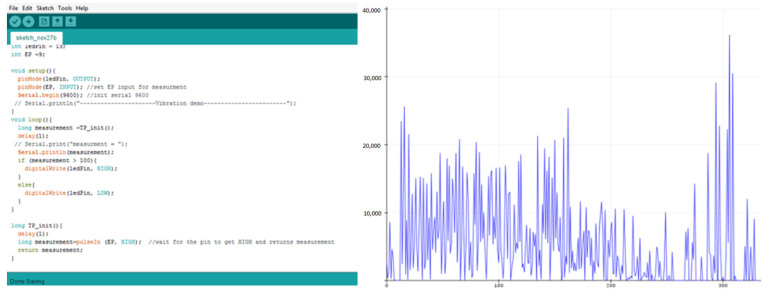
Vibrational frequency in machining of SS304 steels.

To record the vibration behavior with wear, the turning tests were carried out with a vibration sensor attached in the tool holder and the data acquisition system acquires the vibration patterns for both coated and uncoated tool inserts. The experimental setup with SS304 work piece and vibration data acquisition system and vibrational frequency are shown in Figure 1 and Figure 2, respectively.

The combination of spindle speed, depth of cut and feed rate were derived from Taguchi L25 orthogonal array using MINITAB software (Trial version, State College, PA, USA) and 25 combinations of the turning tests were carried out with uncoated Silicon Carbide tool inserts and TiN-coated tool inserts. The combinations of the machining parameters are shown in Table 3.

### 2.2. Numerical Analysis of Vibrational Behavior of Tool Inserts

**Figure 3:**
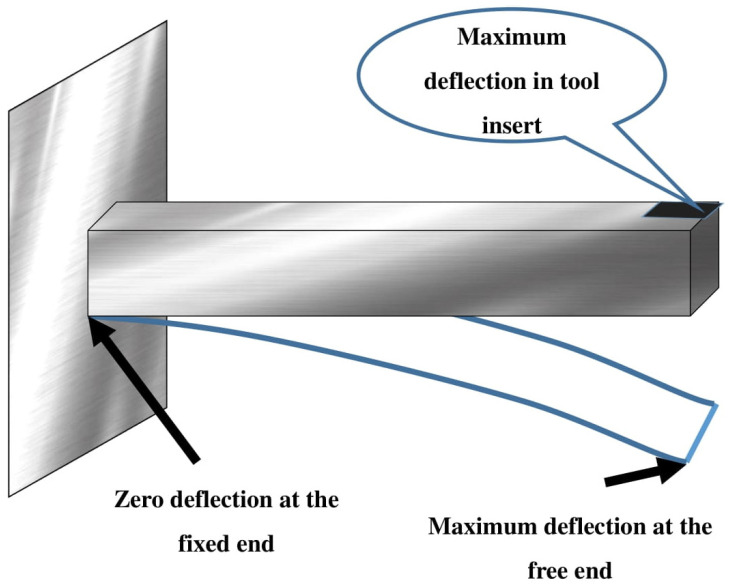
Tool holder as cantilever beam—Finite element method of solving vibrational frequency.

The vibrational behavior of the tool holder was derived numerically by considering the tool holder as cantilever beam [3]. The frequency of vibration was derived by dividing the elements into 10 nodes. The Finite element method was used to solve the governing equations by giving the cutting force as an input parameter. MathCAD prime 7 software (Trial version), was used to compute the nodal equations. In finite element equations, the following assumptions are made to determine the frequency of tool holder and tool insert. A total of 10 Nodes and 9 elements of the cantilever beam tool holder were assumed and exhibited in Figure 3.

Density:


$$\mathrm{Tool holder}\;\rho_{1}=7.8\times 10^{-6}\;\mathrm{kg}/{\mathrm{mm}}^{3}$$

$$\mathrm{Tool insert}\;\rho_{2}=3.210\times 10^{-6}\;\mathrm{kg}/{\mathrm{mm}}^{3}$$

Young’s Modulus:


$$\mathrm{Tool holder}\;E_{1}=2\times 10^{5}\;N/{\mathrm{mm}}^{2}$$

$$\mathrm{Tool inserts}\;E_{2}=4.0138\times 10^{5}\;N/{\mathrm{mm}}^{2}$$

Cutting force = F_c_ = 76.564 N

Length of the tool holder = L_1_ = 10 mm

Length of the tool holder = L_2_ = 5 mm

Height = 10 mm, Width = 10 mm

A_1_ = Width _tool holder_ × height _tool holder_

A_2_ = Width _tool insert_ × height _tool insert_

where A_1_ = 100 mm^2^; A_2_ = 0.2 mm^2^.

where

M1 and M2 are the material property of the tool holder and tool insert, respectively.

‘E_1_’ and ‘E_2_’ are the Young’s modulus of tool holder and tool insert, respectively (N/mm^2^).

‘A_1_’ and ‘A_2_’ are the area of the cross-section of the tool holder and tool insert, respectively (mm^2^).

‘L_1_’ and ‘L_2_’ are the length of the elements for tool holder and tool insert, respectively (mm).

M_1_ = A_1_ × $\frac{E_{1}}{L_{1}}=2\times 10^{6}\;N/m$

M_2_ = A_2_ × $\frac{E_{2}}{L_{2}}=1.0606\times 10^{4}\;N/m$

$C_{2}=\rho_{2}A_{2}\cdot \frac{L_{2}}{2}$

Stiffness matrix:


$$K\;:=\;\left[\begin{array}{ccccccccc} 2*M1 & -M1 & 0 & 0 & 0 & 0 & 0 & 0 & 0\\ -M1 & 2*M1 & -M1 & 0 & 0 & 0 & 0 & 0 & 0\\ 0 & -M1 & 2*M1 & -M1 & 0 & 0 & 0 & 0 & 0\\ 0 & 0 & -M1 & 2*M1 & -M1 & 0 & 0 & 0 & 0\\ 0 & 0 & 0 & -M1 & 2*M1 & -M1 & 0 & 0 & 0\\ 0 & 0 & 0 & 0 & -M1 & 2*M1 & -M1 & 0 & 0\\ 0 & 0 & 0 & 0 & 0 & -M1 & 2*M1 & -M1 & 0\\ 0 & 0 & 0 & 0 & 0 & 0 & -M1 & M1+M2 & -M2\\ 0 & 0 & 0 & 0 & 0 & 0 & 0 & -M2 & M2 \end{array}\right]$$

$$K=\left[\begin{array}{ccccccccc} 4*10^{6} & -2*10^{6} & 0 & 0 & 0 & 0 & 0 & 0 & 0\\ -2*10^{6} & 4*10^{6} & -2*10^{6} & 0 & 0 & 0 & 0 & 0 & 0\\ 0 & -2*10^{6} & 4*10^{6} & -2*10^{6} & 0 & 0 & 0 & 0 & 0\\ 0 & 0 & -2*10^{6} & 4*10^{6} & -2*10^{6} & 0 & 0 & 0 & 0\\ 0 & 0 & 0 & -2*10^{6} & 4*10^{6} & -2*10^{6} & 0 & 0 & 0\\ 0 & 0 & 0 & 0 & -2*10^{6} & 4*10^{6} & -2*10^{6} & 0 & 0\\ 0 & 0 & 0 & 0 & 0 & -2*10^{6} & 4*10^{6} & -2*10^{6} & 0\\ 0 & 0 & 0 & 0 & 0 & 0 & -2*10^{6} & 2.016*10^{6} & -1.606*10^{4}\\ 0 & 0 & 0 & 0 & 0 & 0 & 0 & -1.606*10^{4} & 1.606*10^{4} \end{array}\right]$$

Lumped mass matrix (Consistent matrix):

$$M\;:=\;\left[\begin{array}{ccccccccc} 2*C1 & 0 & 0 & 0 & 0 & 0 & 0 & 0 & 0\\ 0 & 2*C1 & 0 & 0 & 0 & 0 & 0 & 0 & 0\\ 0 & 0 & 2*C1 & 0 & 0 & 0 & 0 & 0 & 0\\ 0 & 0 & 0 & 2*C1 & 0 & 0 & 0 & 0 & 0\\ 0 & 0 & 0 & 0 & 2*C1 & 0 & 0 & 0 & 0\\ 0 & 0 & 0 & 0 & 0 & 2*C1 & 0 & 0 & 0\\ 0 & 0 & 0 & 0 & 0 & 0 & 2*C1 & 0 & 0\\ 0 & 0 & 0 & 0 & 0 & 0 & 0 & C1+C2 & 0\\ 0 & 0 & 0 & 0 & 0 & 0 & 0 & 0 & C2+\mathrm{Fc} \end{array}\right]$$

$$M=\left[\begin{array}{ccccccccc} 0.008 & 0 & 0 & 0 & 0 & 0 & 0 & 0 & 0\\ 0 & 0.008 & 0 & 0 & 0 & 0 & 0 & 0 & 0\\ 0 & 0 & 0.008 & 0 & 0 & 0 & 0 & 0 & 0\\ 0 & 0 & 0 & 0.008 & 0 & 0 & 0 & 0 & 0\\ 0 & 0 & 0 & 0 & 0.008 & 0 & 0 & 0 & 0\\ 0 & 0 & 0 & 0 & 0 & 0.008 & 0 & 0 & 0\\ 0 & 0 & 0 & 0 & 0 & 0 & 0.008 & 0 & 0\\ 0 & 0 & 0 & 0 & 0 & 0 & 0 & 0.004 & 0\\ 0 & 0 & 0 & 0 & 0 & 0 & 0 & 0 & 76.264 \end{array}\right]$$

$$f=R\;\overset{\left(\mathrm{Solve},ɷ,\mathrm{float}\;5\right)}{\to }\;\left[\begin{array}{c} -9323.9\\ 15112.0\\ 14.065\\ -15112.0\\ 30788.0\\ -30788.0\\ 28194.0\\ -31798.0\\ -14.065\\ -28194.0\\ 9323.9\\ -24766.0\\ -20336.0\\ 20336.0\\ 24766.0\\ 3218.9\\ -3218.9\\ 31798.0 \end{array}\right]$$

From the frequency matrix, the frequency of 28.194 KHz is obtained for the cantilever beam (nearer to the actual value). Similarly, for all trials of the Titanium Nitride-coated tool insert and Silicon Carbide tool insert, Lumped mass matrix is determined and it is illustrated in Table 4.

## 3. Results and Discussions

The feed, cutting speed and depth of cut are the machining parameters that are taken in to consideration and the cutting forces in Cartesian coordinates and tool wear are monitored. The flank wear and crater wear are observed in turning tests. The variation in the cutting forces compared to the corresponding machining parameters and depicted in Table 5. The regression equation depicts the significance of flank wear in tool failure. Crater and flank wear for both the SiC tool insert and TiN-coated tool insert exhibits a depth of cut that strongly influences tool failure [18]. The regression for crater wear and flank wear are exhibited in Table 4. The statistical evaluation of errors for experimental values and numerical values are depicted using curve fitting techniques and the results are depicted in Table 4 which portrays the R squared values of experimental vibration frequencies and the Finite element method. The R squared of 81% and 89% are obtained for the experimental and FEM vibrations, respectively. The regression equation correlating machining parameters such as feed rate, speed, and depth of cut shows that wear is the function of machining parameters [19]. The significance of machining parameters in crater wear and flank wear are shown in Table 5.

**Figure 4:**
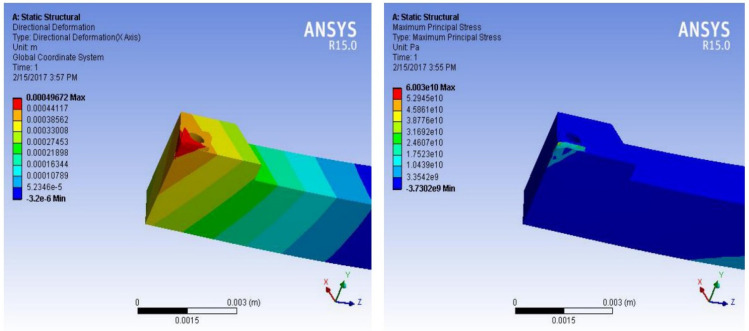
Stress distribution and deformation of tool holder in turning tests.

The visualization of the tool wear and vibrations are depicted in Figure 4 which further showed the tool wear pattern with feed rate, depth of cutting and cutting speed. The stress distribution and deformation are exhibited in Figure 4 The vibration frequencies of SiC and TiN tool inserts shows an increasing trend corresponding to the feed rate, depth of cut and the cutting speed. The amplitude and frequency of the vibration is strongly influenced with the cutting speed and depth of cut, whereas as the feed rate increases, the frequency and amplitude reduces, which shows the damping phenomenon of the tool holder with the work piece.

**Figure 5:**
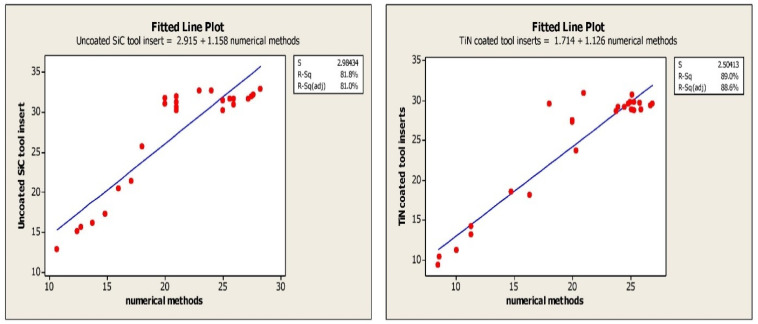
Linear Curve fitting of experimental vibration frequencies and FEM vibration results.

**Figure 6:**
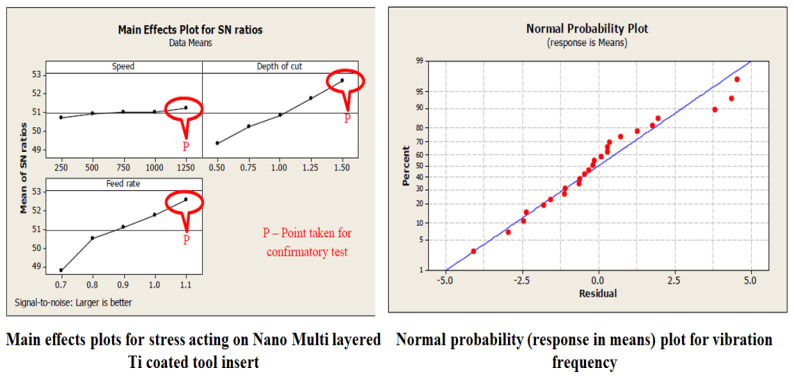
SN ratio and curve fitting for Nano multilayered Titanium-coated tool inserts.

The curve fitting for Coated and Uncoated tool inserts are exhibited in Figure 5. The signal to noise ratio, and curve fitting for coated turning tool inserts are exhibited in Figure 6. The Uncoated turning tool carbide inserts showed the microscopic patches and nucleation of cracks and voids of maximum size crater depth of 0.2 mm. whereas the multilayered Titanium Nitride-coated turning tool inserts showed good wear-resistance properties by 0.07 mm of crater depth and reduction in crack nucleation and voids. Since the wear characteristics of the tool insert is consistent with time, the metal removal time is maintained as constant for both inserts throughout the turning tests. The wear resistance increase produces more cutting forces during the turning operation. This phenomenon leads to a reduction in the stress acting on the tool inserts. The characterization of coating materials defines the behavior of cutting tool stress on the work piece. The stress is calculated based on the cutting forces applied and the cutting tool properties. The finite element technique is adopted to determine the stresses acting on the tool inserts. The flank wear is characterized by the length and crater wear by the width of the workpiece.

**Figure 7:**
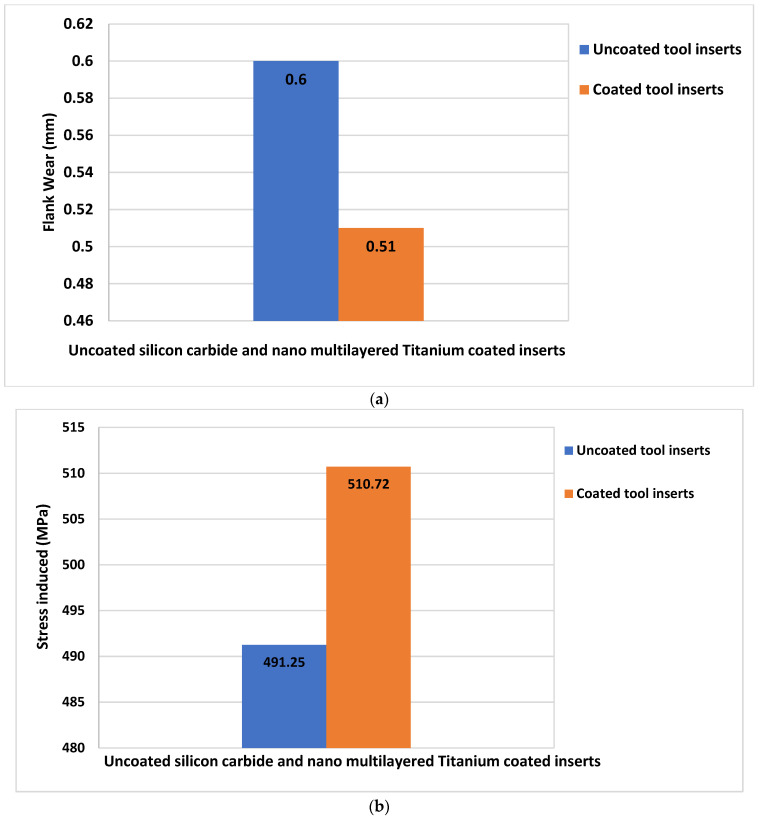
Flank wear and stress characteristics for uncoated and TiN-coated inserts. (**a**) Reduction of flank-wear for uncoated Silicon Carbide and Nano multilayered Titanium tool inserts. (**b**) Improvement of cutting tool stress for uncoated and Nano multilayered Titanium-coated inserts.

**Figure 8:**
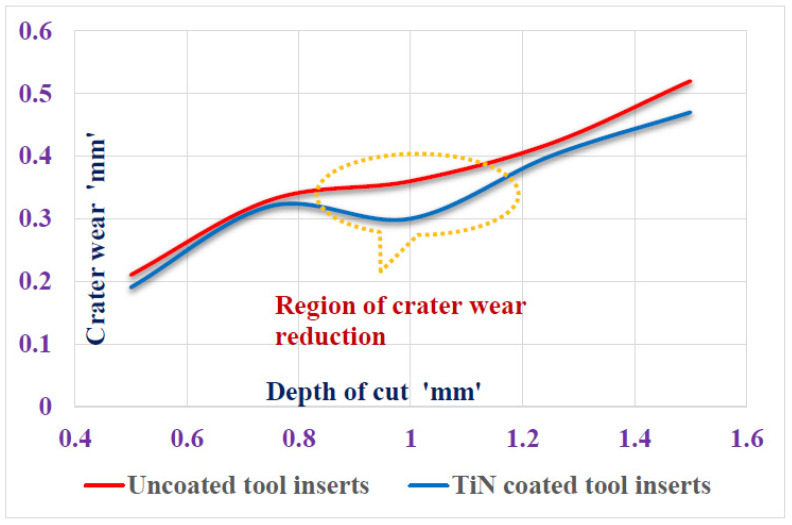
Wear pattern with depth of cut in turning tests.

**Figure 9:**
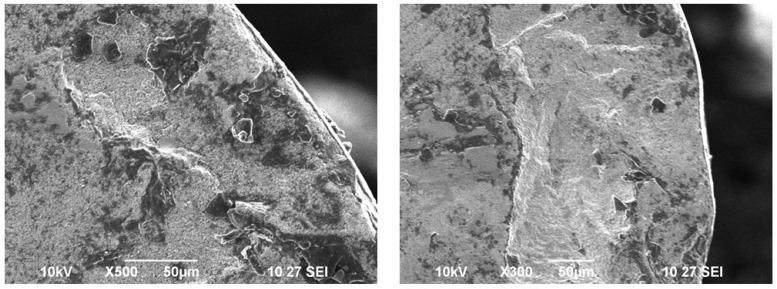
Microscopic images of uncoated and Nano multilayer TiN-coated Silicon Carbide tool inserts.

The variation of flank wear and improvement in cutting tool stress are illustrated in Figure 7a,b. The wear pattern with the machining parameter is illustrated in Figure 8. The wear pattern microscopic images after the turning tests for uncoated Silicon Carbide tool inserts and Titanium Nitride-coated tool inserts are shown in Figure 9.

## 4. Conclusions

Titanium Nitride coating is suitable for machining of SS304 steels and machining characteristics of tool inserts are strongly influenced by the machining parameters such as feed, cutting speed and depth of cut. The vibrations of machine tools affect the quality of machining. The following deductions are performed in the turning tests and FEM results.

1. At a feed rate of 0.7 mm/min, a depth of cut 0.5 mm and a spindle speed of 250 rpm, 12 KHz of vibrational frequency is observed in the uncoated Silicon Carbide tool insert. Meanwhile, in the Titanium Nitride-coated tool insert, the vibration frequency is reduced to 10 KHz;
2. The vibration frequencies of SiC and TiN tool inserts show the increasing trend corresponding to the feed rate, depth of cut and the cutting speed. The amplitude and frequency of the vibration is strongly influenced with the cutting speed and depth of cut, whereas the feed rate increases the frequency and the amplitude reduces which shows the damping phenomenon of the tool holder with the work piece;
3. At the feed rate of 1.1 mm/min, a depth of cut of 1.5 mm and a spindle speed of 1000 rpm, maximum stress is found in the tool insert (both the Silicon Carbide insert and Titanium Nitride-coated insert) contact region of insert and SS304 work piece. For the uncoated Silicon Carbide tool insert, 394.6 MPa of stress is found. Meanwhile, in the Titanium Nitride-coated tool insert, a maximum stress of 620.84 MPa has been observed;
4. The contribution of the flank wear is greater compared to the crater wear. At the feed rate of 1.1 mm/min, depth of cut of 1.5 mm and spindle speed of 1000 rpm, 60% of flank wear and 40% of crater wear are contributed by the tool size reduction;
5. The wear pattern microscopic images taken after the turning tests unveiled that the uncoated tungsten carbide inserts reported the microscopic patches and nucleation of cracks as well as voids of maximum size and crater depth of 0.2 mm. As a result, multilayered Titanium Nitride-coated turning tool inserts have shown superior wear resistance by reducing crack nucleation and voids by 0.07 mm.

## Figures and Tables

**Table 1:** Machining parameters for turning tests.

Feed Rate mm/rev	Depth of Cut mm	Speed rpm
0.7	0.5	250
0.8	0.75	500
0.9	1	750
1	1.25	1000
1.1	1.5	1250

**Table 2:** Tool Nomenclature for CNMG turning tool holder.

End cutting edge angle	−5°
Back rake angle	−5°
Side rake angle	5°
Side relief angle	10°
End relief angle	10°
Nose radius	0.7 mm

**Table 3:** Taguchi L25 orthogonal array—Combination of machining parameters.

Trials	Depth of Cut	Speed	Feed Rate
	mm	rpm	mm/min
1	0.5	250	0.19
2	0.75	500	0.29
3	1	750	0.3
4	1.25	1000	0.39
5	1.5	1250	0.46
6	0.5	500	0.24
7	0.75	750	0.36
8	1	1000	0.4
9	1.25	1250	0.48
10	1.5	250	0.57
11	0.5	750	0.35
12	0.75	1000	0.44
13	1	1250	0.53
14	1.25	250	0.59
15	1.5	500	0.69
16	0.5	1000	0.45
17	0.75	1250	0.59
18	1	250	0.61
19	1.25	500	0.64
20	1.5	750	0.76
21	0.5	1250	0.55
22	0.75	250	0.68
23	1	500	0.72
24	1.25	750	0.75
25	1.5	1000	0.84

**Table 4:** Vibration pattern and wear of cutting tools in turning tests.

	Vibration Frequency				Tool Wear			
	Experimental		Numerical Analysis		Flank Wear		Crater Wear	
Trials	Uncoated SiC Tool Insert	TiN-Coated Tool Insert	Uncoated SiC Tool Insert	TiN-Coated Tool Insert	Flank Wear Uncoated Tool Inserts	Flank Wear Coated Tool Inserts	Crater Wear Uncoated Tool Inserts	Crater Wear Coated Tool Inserts
	KhZ	KhZ	KhZ	KhZ	mm	mm	mm	mm
1	16.123	10.35	13.70	8.59	0.3	0.29	0.2	0.19
2	17.23	11.15	14.82	10.04	0.5	0.49	0.3	0.29
3	12.84	9.31	10.66	8.47	0.58	0.53	0.35	0.3
4	15.56	13.15	12.76	11.31	0.64	0.63	0.4	0.39
5	15.12	14.14	12.40	11.31	0.74	0.7	0.5	0.46
6	20.42	18.13	17.36	16.32	0.54	0.48	0.3	0.24
7	21.34	18.45	17.07	14.76	0.64	0.6	0.4	0.36
8	25.64	23.65	20.51	20.34	0.69	0.64	0.45	0.4
9	31.25	29.53	26.25	26.87	0.74	0.72	0.5	0.48
10	31.65	29.62	27.22	25.77	0.84	0.81	0.6	0.57
11	30.15	29.13	25.33	24.47	0.68	0.63	0.4	0.35
12	32.15	30.65	27.65	25.13	0.78	0.72	0.5	0.44
13	32.65	29.16	26.77	23.91	0.83	0.81	0.55	0.53
14	32.64	30.86	27.09	25.92	0.88	0.87	0.6	0.59
15	30.89	27.46	25.95	23.89	0.98	0.97	0.7	0.69
16	30.15	27.25	25.63	24.25	0.82	0.77	0.5	0.45
17	31.97	28.75	27.49	25.30	0.92	0.91	0.6	0.59
18	31.42	29.58	26.08	26.92	0.97	0.93	0.65	0.61
19	30.55	28.85	25.97	25.10	1.02	0.96	0.7	0.64
20	30.95	28.65	26.00	23.78	1.12	1.08	0.8	0.76
21	31.75	28.79	26.67	25.91	0.96	0.91	0.6	0.55
22	31.95	29.35	27.16	26.71	1.06	1.04	0.7	0.68
23	31.64	29.79	25.94	25.32	1.11	1.08	0.75	0.72
24	31.63	29.75	25.62	24.99	1.16	1.11	0.8	0.75
25	32.89	29.54	28.29	24.81	1.26	1.2	0.9	0.84

**Table 5:** Regression Equations for crater and flank wear.

**Crater Wear**	**Without Coating**	Crater Wear = −0.526 + 0.178 Depth of cut + 0.701 Feed rate + 0.000001 Speed + 0.00083 Fx + 0.00101 Fy + 0.00534 Fz
	**With Coating**	Crater Wear = −0.633 + 1.06 Feed rate + 0.327 Depth of cut − 0.000013 Speed − 0.00273 Fx − 0.0108 Fy + 0.0105 Fz
**Flank Wear**	**Without Coating**	Flank wear = −0.784 + 0.356 Depth of cut + 1.58 Feed rate + 0.000008 Speed − 0.00823 Fx + 0.00289 Fy + 0.00107 Fz
	**With Coating**	Flank wear = −0.807 + 1.54 Feed rate + 0.375 Depth of cut + 0.000029 Speed- 0.00600 Fx − 0.0181 Fy + 0.0199 Fz

## Data Availability

Not applicable.

## References

[1] Lim G.H. (1995). Tool-wear monitoring in machine turning. J. Mater. Process. Technol..

[2] Song D.Y., Otani N., Aoki T., Kamakoshi Y., Ohara Y., Tamaki H. (2005). A new approach to cutting state monitoring in end-mill machining. Int. J. Mach. Tools Manuf..

[3] Liang S.Y., Dornfeld D.A. (1989). Tool wear detection using time series analysis of acoustic emission. J. Eng. Ind..

[4] PalDey S.C.D.S., Deevi S.C. (2003). Single layer and multilayer wear resistant coatings of (Ti, Al) N: A review. Mater. Sci. Eng. A.

[5] Ghani J.A., Choudhury I.A., Masjuki H.H. (2004). Wear mechanism of TiN coated carbide and uncoated cermets tools at high cutting speed applications. J. Mater. Process. Technol..

[6] Özel T., Hsu T.K., Zeren E. (2005). Effects of cutting edge geometry, workpiece hardness, feed rate and cutting speed on surface roughness and forces in finish turning of hardened AISI H13 steel. Int. J. Adv. Manuf. Technol..

[7] Ganeshkumar S., Thirunavukkarasu V., Sureshkumar R., Venkatesh S., Ramakrishnan T. (2019). Investigation of wear behaviour of silicon carbide tool inserts and titanium nitride coated tool inserts in machining of EN8 steel. Int. J. Mech. Eng. Technol..

[8] Kumar S.G., Thirunavukkarasu V. (2016). Investigation of Tool Wear and Optimization of Process Parameters in Turning of EN8 and EN 36 Steels. Asian J. Res. Soc. Sci. Humanit..

[9] De Oliveira V.V., Beltrão P.D.C., Pintaude G. (2011). Effect of tool geometry on the wear of cemented carbide coated with TiAlN during drilling of compacted graphite iron. Wear.

[10] Lane B.M., Shi M., Dow T.A., Scattergood R. (2010). Diamond tool wear when machining Al6061 and 1215 steel. Wear.

[11] Attanasio A., Ceretti E., Fiorentino A., Cappellini C., Giardini C. (2010). Investigation and FEM-based simulation of tool wear in turning operations with uncoated carbide tools. Wear.

[12] Vijayaraghavan V., Garg A., Gao L., Vijayaraghavan R., Lu G. (2016). A finite element based data analytics approach for modeling turning process of Inconel 718 alloys. J. Clean. Prod..

[13] Venkatesh S., Sivapirakasam S.P., Sakthivel M., Ganeshkumar S., Prabhu M.M., Naveenkumar M. (2021). Experimental and numerical investigation in the series arrangement square cyclone separator. Powder Technol..

[14] Ganeshkumar S., Sureshkumar R., Sureshbabu Y., Balasubramani S. (2019). A Numerical Approach to Cutting Tool Stress in CNC Turning of En8 Steel with Silicon Carbide Tool Insert. Int. J. Sci. Technol. Res..

[15] Ganeshkumar S., Sureshkumar R., Sureshbabu Y., Balasubramani S. (2020). A Review On Cutting Tool Measurement in Turning Tools by Cloud Computing Systems in Industry 4.0 and IoT. GIS Sci. J..

[16] Liu E., Wang R., Zhang Y., An W. (2021). Tool wear analysis of cutting Ti-5553 with uncoated carbide tool under liquid nitrogen cooling condition using tool wear maps. J. Manuf. Process..

[17] Ganeshkumar S., Kumar S.D., Magarajan U., Rajkumar S., Arulmurugan B., Sharma S., Li C., Ilyas R.A., Badran M.F. (2022). Investigation of Tensile Properties of Different Infill Pattern Structures of 3D-Printed PLA Polymers: Analysis and Validation Using Finite Element Analysis in ANSYS. Materials.

[18] Ghule G.S., Sanap S., Adsul S., Chinchanikar S., Gadge M. (2022). Experimental investigations on the ultrasonic vibration-assisted hard turning of AISI 52100 steel using coated carbide tool. Mater. Today Proc..

[19] Stephenson D.A., Agapiou J.S. (2006). Metal Cutting Theory and Practice.

